# Naturally Occurring Anthraquinones: Chemistry and Therapeutic Potential in Autoimmune Diabetes

**DOI:** 10.1155/2015/357357

**Published:** 2015-03-18

**Authors:** Shih-Chang Chien, Yueh-Chen Wu, Zeng-Weng Chen, Wen-Chin Yang

**Affiliations:** ^1^Department of Forestry, National Chung-Hsing University, Taichung 402, Taiwan; ^2^Agricultural Biotechnology Research Center, Academia Sinica, No. 128, Academia Sinica Road, Sec. 2, Nankang, Taipei 115, Taiwan; ^3^Animal Technology Institute, Chunan 350, Taiwan; ^4^Department of Life Sciences, National Chung-Hsing University, Taichung 402, Taiwan; ^5^Institute of Biotechnology, National Taiwan University, Taipei 106, Taiwan; ^6^Department of Aquaculture, National Taiwan Ocean University, Keelung 202, Taiwan; ^7^Institute of Pharmacology, Yang-Ming University, Taipei 112, Taiwan

## Abstract

Anthraquinones are a class of aromatic compounds with a 9,10-dioxoanthracene core. So far, 79 naturally occurring anthraquinones have been identified which include emodin, physcion, cascarin, catenarin, and rhein. A large body of literature has demonstrated that the naturally occurring anthraquinones possess a broad spectrum of bioactivities, such as cathartic, anticancer, anti-inflammatory, antimicrobial, diuretic, vasorelaxing, and phytoestrogen activities, suggesting their possible clinical application in many diseases. Despite the advances that have been made in understanding the chemistry and biology of the anthraquinones in recent years, research into their mechanisms of action and therapeutic potential in autoimmune disorders is still at an early stage. In this paper, we briefly introduce the etiology of autoimmune diabetes, an autoimmune disorder that affects as many as 10 million worldwide, and the role of chemotaxis in autoimmune diabetes. We then outline the chemical structure and biological properties of the naturally occurring anthraquinones and their derivatives with an emphasis on recent findings about their immune regulation. We discuss the structure and activity relationship, mode of action, and therapeutic potential of the anthraquinones in autoimmune diabetes, including a new strategy for the use of the anthraquinones in autoimmune diabetes.

## 1. Autoimmune Diabetes

### 1.1. Etiology and Therapies for Autoimmune Diabetes

Autoimmune diabetes (AID) is a life-threatening metabolic disease that is initiated and progresses through a complex interplay of environmental, genetic, and immune factors. As a result, insulin-producing *β*-cells are destroyed by leukocytes leading to insufficient/deficient insulin that fails to maintain blood glucose homeostasis, and lethal macro- and microvascular complications ensue. In 2013, the International Diabetes Federation (IDF) estimated that some 79,000 children under 15 years develop AID annually worldwide [1].

In patients and animal models of AID, at disease onset, leukocytes infiltrate into the pancreatic islets [2]. Among the leukocytes, T lymphocytes are the main players in AID although B lymphocytes, dendritic cells, macrophages, and NK cells are also implicated in this invasion, a condition termed insulitis [3, 4]. This invasion contributes to a gradual loss of pancreatic *β*-cells, leading to insulin insufficiency/deficiency and then hyperglycemia, two hallmarks of AID [5].

**Figure 1:**
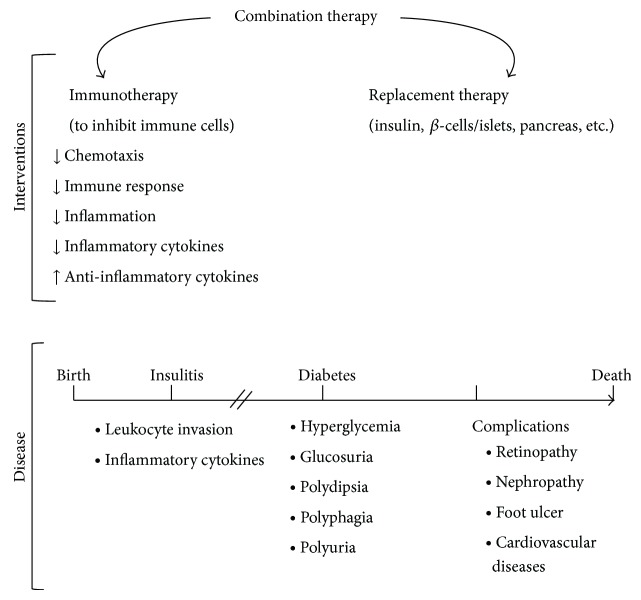
AID development and intervention. During AID onset, leukocytes start to invade pancreatic islets, a condition termed insulitis, followed by diabetes. Diabetes is characterized by hyperglycemia, insulin insufficiency/deficiency, and glucosuria. Polydipsia, polyphagia, and polyuria are found in diabetic patients. Diabetic complications such as retinopathy, nephropathy, foot ulcers, and cardiovascular disease result in fatality of patients. Immunotherapy, replacement therapy, and combinations of both are common approaches to treat AID.

So far, insulin injection is the only way to control AID; however, it fails to cure the disease and can only ameliorate its complications. Therefore, discovery of novel and effective approaches to cure AID is necessary. Immune therapy, replacement therapy using insulin, *β*-cells, islets, and pancreas, and combination therapy have all been tested to prevent and treat AID (Figure 1) [6]. Migration of leukocytes during diabetes development is viewed as a critical target through which to interfere with the disease onset and progression. From the immune perspective, chemokines and their pathways are attractive targets for intervention and may hold the key to stopping insulitis and, thus, delay or prevent AID [7–10]. Preservation of functional *β*-cells is equally crucial for curing AID [11]. This topic has been reviewed elsewhere [12], however, and is not within the scope of this paper.

### 1.2. Chemotaxis and Its Mechanism in Leukocytes

**Figure 2:**
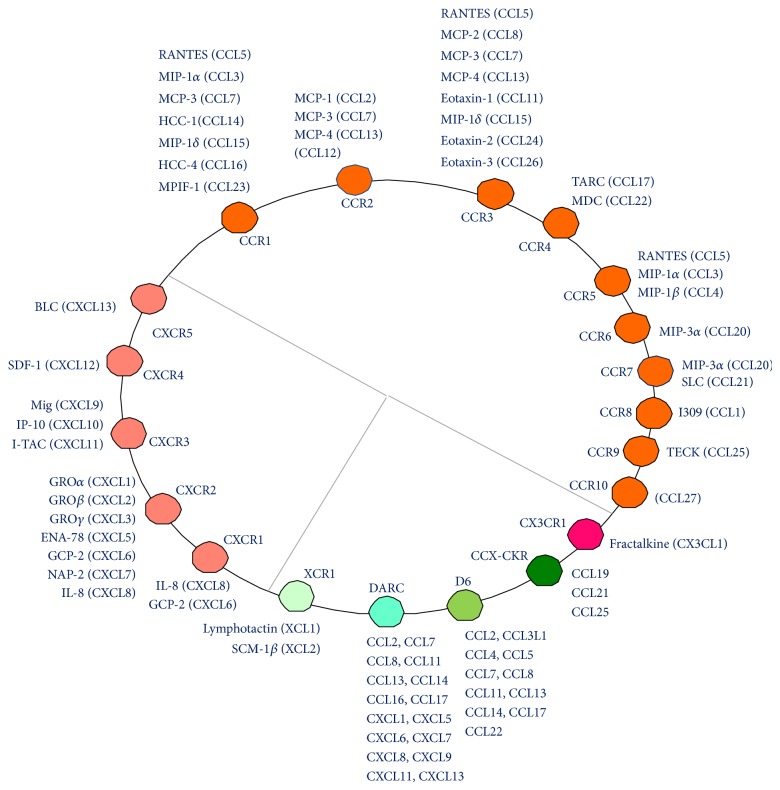
Chemokines and their cognate receptors. Twenty-three chemokine receptors and their natural ligands are classified into CCR, CXCR, and other categories.

In mammals, 23 chemokine receptors and over 50 chemokines have been discovered (Figure 2) [13]. They function in health and disease in roles such as cell recruitment during embryogenesis, leukocyte trafficking, helper T cell differentiation, angiogenesis, HIV infection, sepsis, atherosclerosis, inflammation, immune disorders, and cancer metastasis [14]. One of the most important functions of chemokine/chemokine receptors is to direct the migration of leukocytes from the venous system to sites of inflammation. They play an essential role in inflammation and, as a consequence, inflammatory diseases such as autoimmune diseases and cancers [15]. Structurally speaking, chemokine receptors belong to a family of 7-helix transmembrane G protein-coupled receptors (GPCRs). Upon chemokine engagement, chemokine receptors initiate the binding of the G*α* subunit to guanosine triphosphate and the dissociation of the G*α* subunit from the G*βγ* subunit. This activates protein tyrosine kinases, mitogen-activated protein (MAP) kinases, and phospholipase C. Secondary messengers, inositol triphosphate and diacylglycerol, which are converted from phosphatidylinositol by phospholipase C, induce cellular calcium influx and translocation/activation of protein kinase C, respectively. The above biochemical cascades lead to cell chemotaxis and other cell functions (Figure 4(a)) [16]. Hence, chemokines/chemokine receptors have been proposed as drug targets for inflammatory diseases [14, 17–19]. For instance, the first FDA approved CXCR4 antagonist, plerixafor/AMD3100, is used to mobilize hematopoietic stem cells, which are collected for use in stem cell graft in patients with hematological cancers. Plerixafor was initially developed to interfere with SDF-1/CXCR4 interaction and shows promise for HIV infection, cancers, and autoimmune diseases such as rheumatoid arthritis [20]. However, this drug is expensive because of the difficulty in its total synthesis. There is, therefore, a demand for the discovery of new CXCR4 antagonists that are both cost-effective and potent.

Since T cells and other leukocytes are thought to be essential players in AID [3, 21], interference with chemokine receptors in leukocytes could be a promising approach for treating insulitis and AID prophylaxis. CXCR4 is expressed in all the leukocytes including naïve T cells [22]. CCR5 is preferentially expressed in activated T cells and macrophages [23–25]. And CCR3 and CCR4 are implicated in Th2 cells whereas CXCR3 and CCR5 are associated with Th1 cells [14]. On the flip side, genetic studies further showed that deficiency in CXCR3 and CCR2 accelerated AID in NOD mice [26, 27]. In contrast, CCR5 ablation delayed AID [27], which was contradictory to one publication indicating that CCR5 positively regulated AID [28]. Anti-CXCL10 was reported to delay AID in NOD mice, implying that CXCR3 may accelerate AID [29]. Overexpression of D6 in pancreatic islets reduced AID in NOD mice [30]. Overexpression of CCL2, a natural ligand for DARC, D6, and CCR2, in the pancreas reduced AID in NOD mice [31], which is consistent with a negative regulation of AID by CCR2, D6, and DACR. Of them, the impact of DARC in AID is unclear.

### 1.3. Mouse Models of AID

Animal models are indispensable for dissecting pathogenesis and for preclinical trials in AID despite some difference between animal models and patients. The animal models include streptozotocin- (STZ-) treated mice, nonobese diabetic (NOD) mice, Biobreeding (BB) rats, Long Evans Tokushima Lean (LETL) rats, New Zealand white rabbits, Chinese hamsters, Keeshond dogs, and Celebes black apes [12].

## 2. Naturally Occurring Anthraquinones

### 2.1. Chemical Structure and Biosynthesis of Naturally Occurring Anthraquinones

**Figure 3:**
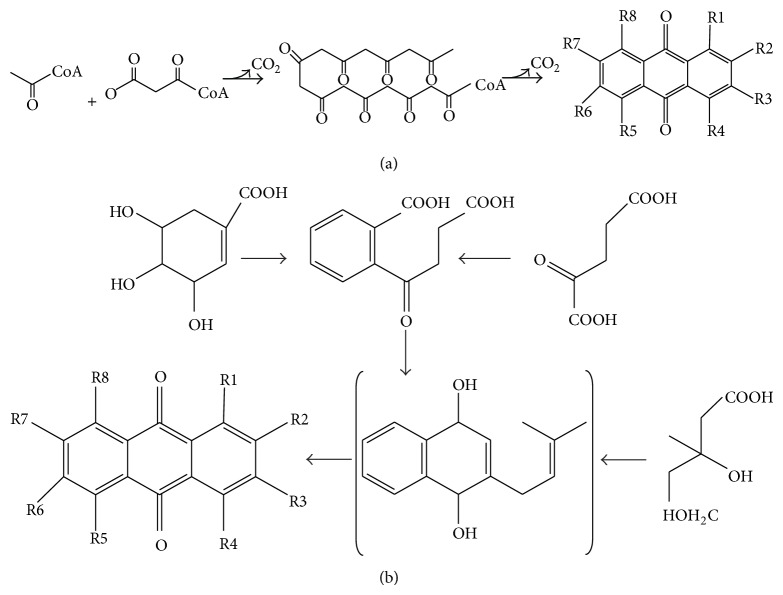
Schema outlining the biosynthesis of anthraquinones. Anthraquinones can be synthesized from acetyl CoA and malonyl CoA via the polyketide pathway (a), or from shikimic acid (b) via the shikimate pathway.

**Figure 4:**
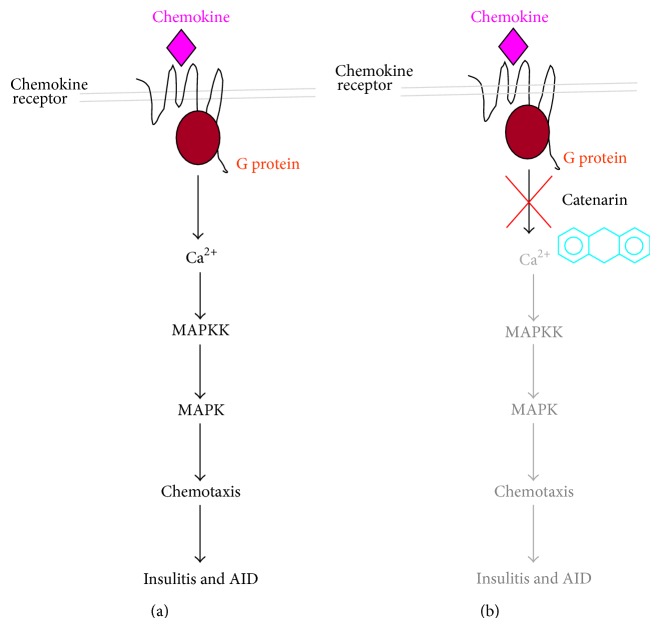
Mode of action of catenarin and other anthraquinones for AID. (a) Upon chemokine binding, a chemokine receptor is activated and induces G protein activation. A cascade of calcium mobilization and activation/phosphorylation of MAPKK/MAPK pathways leads to chemotaxis of leukocytes and, subsequently, insulitis and diabetes. (b) Catenarin and probably other anthraquinones inhibit leukocyte migration mediated by CCR5 and CXCR4 via the inactivation of MAPKs (p38 and JNK), MKKs (MKK6 and MKK7), and calcium mobilization. As a result, anthraquinones can suppress insulitis and diabetes.

Naturally occurring anthraquinones (NOAQs) are a group of secondary metabolites structurally related to 9,10-dioxoanthracene (also known as anthracene 9,10-diones) and their glycosides (Table 1 and Figure 4). Currently, there are 79 known NOAQs [32], which were isolated from lichens, fungi, or higher medicinal plants (e.g., Polygonaceae, Rhamnaceae, Rubiaceae, Fabaceae, and Xanthorrhoeaceae) [32–38]. Although their biosynthetic pathways are not yet fully clear, NOAQs can be biosynthesized from the polyketide (Figure 3(a)) or shikimate (Figure 3(b)) pathway as described in Figure 3 [39]. They can be formed either by the cyclization of linear octa-*β*-ketoacyl CoA intermediates from the addition of one acetyl CoA to three malonyl CoA or by the addition of succinoylbenzoic acid, resulting from shikimic acid and*α*-ketoglutaric acid, to mevalonic acid.

### 2.2. Mechanism of Action of NOAQs in AID

NOAQs have widespread applications throughout medicine as well as in industry. Medicinally speaking, they show a wide spectrum of bioactivities. Most of them are best known as laxative compounds for constipation. Apart from laxative activity, emodin, the most studied anthraquinone, has been reported to have cathartic, anti-inflammatory, anticancer, antimicrobial, diuretic, DNA-binding, and vasorelaxant activities [2, 55–57]. In addition, emodin, physcion, anthraglycoside B, citreorosein, and emodin 8-O-*β*-D-glucopyranoside were found to have laxative, anti-inflammatory, and other activities [58–60]. Emodin and physcion are kinase and tyrosinase inhibitors [61–63] and also show cytotoxicity against cancer cells [55, 64]. In addition, emodin, citreorosein, and emodin 8-O-*β*-D-glucopyranoside showed phytoestrogen activity [60, 65, 66]. Further, anthraglycoside B has been used to treat acute hepatitis and leukocyte reduction [59].

Recently, NOAQs have been explored for their potential in AID intervention. In one study,* Fallopia japonica*, an anti-inflammatory herb rich in anthraquinones, was tested for AID. NOD mice received intraperitoneal injection of the* F. japonica* crude extract at 40 *μ*g/kg BW, 3 times a week from 4 to 30 weeks (prevention) or from 9 to 30 weeks (therapy). Twelve-week-old NOD mice started to develop AID, and 100% of NOD mice aged 24 weeks and above developed AID. Remarkably, 86% and 80% of 30-week-old NOD mice treated with* F. japonica* crude extract did not develop AID [67]. Consistent with AID incidence, the crude extract delayed and reduced the invasion of leukocytes into the pancreatic islets [67]. Using a chemotaxis-based fractionation and isolation approach, two anthraquinones, emodin and physcion, were isolated and identified from this plant [67]. Moreover,* in vitro* study showed that emodin and physcion exhibited a reduction in CXCR4-mediated migration of Jurkat cells, a human T cell line [67]. A negative control, resveratrol, had no effect on the CXCR4-implicated migration. This reduction in migration involved the inhibition of MAPKs, ERK 1/2, and MAPKK, MEK 1/2 [67]. Accordingly, at doses from 4 mg/kg to 40 mg/kg, emodin and physcion dose-dependently reduced insulitis and AID in NOD mice [67].

In another study, Shen and colleagues showed that catenarin, cascarin, emodin, and rhein inhibited CXR4-mediated chemotaxis in Jurkat T cells [28]. The half maximal inhibitory concentrations (IC_50_) of the CXCR4-mediated migration for catenarin, cascarin, emodin, and rhein were 0.18 *μ*g/mL, 0.3 *μ*g/mL, 0.3 *μ*g/mL, and 2.7 *μ*g/mL, respectively [28]. The IC_50_ of the CCR5-mediated migration for catenarin, emodin, cascarin, and rhein were 0.5 *μ*g/mL, 0.75 *μ*g/mL, 1.46 *μ*g/mL, and 2.5 *μ*g/mL, respectively [28]. Catenarin had higher antichemotactic activity than the other anthraquinones. This activity appears to relate to the number of hydroxyl groups at R5 and R7 in the anthraquinones, revealing a structure-activity relationship of hydroxyl groups in anthraquinones. The *μ*-slide assays, used to follow the trafficking direction, also demonstrated that, at a dose of 0.5 *μ*g/mL, catenarin could completely stop cell movement towards the specific chemokine gradient [28]. These data suggest that the potential of the anthraquinones to inhibit chemotaxis depends on chemokine properties [28]. Further, in an* in vivo* study, 100% of NOD mice developed AID at the age of 24 weeks or beyond. The diabetic incidence of NOD mice treated with catenarin at 0.4, 4, and 20 mg/kg, 3 times a week, from 4 to 30 weeks, was 80%, 70%, 30%, and 0%. NOD mice treated with 20 mg/kg catenarin had normal blood glucose (<200 mg/dL) and Hb_*A1c*_ (<4%), intact islet structure, and very few leukocytes (CD4^+^ T, CD8^+^ T, dendritic cells, macrophages, and NK and B cells) in the pancreatic islets [28]. Flow cytometry showed that catenarin did not influence the expression of chemokine receptors on the cell surface, excluding the possibility that catenarin (and, probably, other anthraquinones) work(s) at the level of the chemokine receptors [28]. In addition, catenarin reduced calcium mobilization in Jurkat cells whilst being exposed to CXCR4 ligand, SDF-1, and CCR4 ligand, MIP-1 [28]. Further, catenarin inhibited JNK and p38 but not ERK 1/2 and, in turn, their upstream regulators, MKK 6/7 [28]. These mechanistic studies concluded that catenarin and/or its derivatives exerted antidiabetic action via chemotactic regulation of leukocytes involving the Ca^2+^/MAPKK/MAPK pathways (Figure 4(b) and Table 2). Of note, catenarin has the highest antichemotactic activity, followed in decreasing order by emodin, cascarin, and rhein. Interestingly, catenarin has two hydroxyl groups at R4 and R6 in its anthraquinone ring. Emodin has only one hydroxyl group at R6. Cascarin and rhein have no hydroxyl groups at R4 and R6. This activity seems to be related to the number of hydroxyl groups at R4 and R6 in anthraquinones as described in Figure 4(a) and Table 1.

Diacerein is a commercial drug commonly utilized to treat human osteoarthritis. It was developed from its prodrug rhein. Very interestingly, diacerein can be used to treat AID in NOD mice [68] akin to its prodrug, rhein [28]. Overall, the data supported the notion that rhein in its stable form, albeit at low efficacy, may be a better pharmaceutical intervention than the other anthraquinones with higher activity.

There is lack of information about the impact of the other chemokines/chemokine receptors in AID. We showed that NOAQs can target CXCR4 and CCR5 pathways [28, 67]. CXCR3, CCR2, CXCL10, CCL2, and D6 were reported to control diabetes development [26, 27, 29–31]. Whether NOAQs target their pathways remains unclear.

## 3. Toxicology

Emodin, one of the most well-studied anthraquinones, is frequently present in laxative herbs. Furthermore, emodin is reported to be effective against cancer, constipation, inflammation, microbes, and peptic ulcers [69]. However, its safety and effectiveness in naturopathic treatment have not been approved by the U.S. Food and Drug Administration (FDA). Side effects of emodin, and probably other anthraquinones, include potential carcinogenesis, nausea, diarrhea, and renal failure. Two anthraquinone-type agents, danthron, a drug for constipation, and diacerein, an anti-inflammatory drug for osteoarthritis, were developed and approved by the U.S. FDA [68, 70]. However, danthron was withdrawn by the FDA in 1999 due to the risk of carcinogenesis [70]. Therefore, clinical use of the anthraquinones should be considered cautiously.

## 4. Conclusions and Perspectives

Several NOAQs show anti-inflammatory activity. Among them, cascarin, catenarin, rhein, physcion, and emodin suppress the chemotactic activity of leukocytes at the insulitis stage of AID development. They suppress chemokine-mediated leukocyte migration towards pancreatic islets leading to a decline in AID development. This suppression involves anthraquinone-mediated inhibition of MAPKK/MAPK pathway. An antiosteoarthritic anthraquinone drug, diacerein, has been shown to prevent AID in a NOD model, suggesting that the antichemotactic activity of the risk-free anthraquinones can likely be exploited for AID and other inflammatory diseases.

## Figures and Tables

**Table 1:** Chemical structure of NOAQs in different plants.

S. number	IUPAC names	Structure								Species
		R1	R2	R3	R4	R5	R6	R7	R8	
1	Tectoquinone (2-methyl-AQ)	H	Me	H	H	H	H	H	H	*Prismatomeris malayana* [40], *Rubia cordifolia* [41],* Rubia oncotricha* [32],and* Rubia tinctorum* [42]
2	2-(Hydroxymethyl)-AQ	H	HOCH_2_	H	H	H	H	H	H	*Rubia yunnanensis* [43] and *Rubia tinctorum* [44]
3	2-Methoxy-AQ	H	MeO	H	H	H	H	H	H	*Rubia tinctorum* [42]
4	2-Hydroxy-AQ	H	OH	H	H	H	H	H	H	*Rubia tinctorum* [42]
5	1-Methoxy-AQ	OH	H	H	H	H	H	H	H	*Rubia cordifolia* [45, 46]
6	1-Hydroxy-2-methyl AQ	OH	Me	H	H	H	H	H	H	*Rubia tinctorum* [42]*, Rubia cordifolia* [46]*, Rubia akane* [39]*, Rubia lanceolata* [39]*, Rubia oncotricha* [39], *Rubia sylvatica* [39], and* Rubia yunnanensis* [43]
7	1-Hydroxy-2-(hydroxymethyl)-AQ	OH	HOCH_2_	H	H	H	H	H	H	*Rubia cordifolia* [32]
8	2-(Ethoxycarbonyl)-1-hydroxy-AQ	OH	EtOOC	H	H	H	H	H	H	*Rubia akane* [39]
9	1-Methoxy-2-methyl-AQ	MeO	Me	H	H	H	H	H	H	*Rubia tinctorum *
10	Alizarin (1,2-dihydroxy-AQ)	OH	OH	H	H	H	H	H	H	*Rubia lanceolata* [39],* Rubia akane* [39], and* Galium sinaicum* [47]
11	Alizarin 2-methyl ether (1-hydroxy-2-methoxy-AQ)	OH	MeO	H	H	H	H	H	H	*Rubia tinctorum* [42]*, Rubia oncotricha* [39],* Rubia cordifolia* [32], and *Galium sinaicum* [47]
12	Alizarin 1-methyl ether (2-hydroxy-1-methoxy-AQ)	MeO	OH	H	H	H	H	H	H	*Rubia tinctorum* [42]
13	Alizarin 1,2-dimethyldiether (1,2-dimethoxy-AQ)	MeO	MeO	H	H	H	H	H	H	*Rubia tinctorum* [42]
14	Rubiadin (1,3-dihydroxy-2- methyl-AQ)	OH	MeO	OH	H	H	H	H	H	*Prismatomeris malayana* [40]*, Rubia tinctorum* [42]*, Rubia cordifolia* [32], and* Rubia lanceolata* [39],* Rubia yunnanensis* [43]
15	Lucidin (1,3-dihydroxy-2- (hydroxymethyl)-AQ)	OH	HOCH_2_	OH	H	H	H	H	H	*Rubia cordifolia* [32]*, Rubia tinctorum* [42], and* Rubia iberica* [33]
16	Nordamnacanthal (1,3-dihydroxy-2-formyl-AQ)	OH	CHO	OH	H	H	H	H	H	*Rubia cordifolia* [32] and * Rubia iberica* [33]
17	Munjistin (1,3-dihydroxy-2-carboxy-AQ)	OH	HOOC	OH	H	H	H	H	H	*Rubia tinctorum* [42]
18	1,3-Dihydroxy-2-(methoxycarbonyl)-AQ	OH	MeOOC	OH	H	H	H	H	H	*Rubia tinctorum* [32]
19	2-(Ethoxymethyl)-1,3-dihydroxy-AQ	OH	EtOCH_2_	OH	H	H	H	H	H	*Rubia cordifolia* [32]
20	1,3-Dihydroxy-2-(methoxymethyl)-AQ	OH	MeOCH_2_	OH	H	H	H	H	H	*Rubia cordifolia* [32]
21	Lucidin dimethyl ether	MeOH	HOCH_2_	MeO	H	H	H	H	H	*Rubia lanceolata* [39]
22	Munjistin dimethyl ether (2-carboxy- 1,3-dimethoxy-AQ)	MeOH	HOOC	MeO	H	H	H	H	H	*Rubia cordifolia* [32]
23	2-Benzylxanthopurpurin	OH	PhCH_2_	OH	H	H	H	H	H	*Rubia tinctorum* [32]
24	Anthragallol 3-methyl ether	OH	OH	MeO	H	H	H	H	H	*Rubia tinctorum* [42]
25	Anthragallol 2,3-dimethyl ether	OH	MeO	MeO	H	H	H	H	H	*Rubia tinctorum* [32]
26	2-Carboxy-1-hydroxy-3-methoxy-AQ	OH	HOOC	MeO	H	H	H	H	H	*Rubia cordifolia* [48]
27	3-Hydroxy-1-methoxy-2-(methoxymethyl)-AQ	MeO	MeOCH_2_	OH	H	H	H	H	H	*Rubia cordifolia* [49]
28	Anthragallol (1,2,3-trihydroxy-AQ)	OH	OH	OH	H	H	H	H	H	*Rubia tinctorum* [32]
29	Purpurin (1,2,4-trihydroxy AQ)	OH	OH	H	OH	H	H	H	H	*Rubia tinctorum* [32, 42]*, Rubia cordifolia* [32]*, Rubia munjista* [33]*, Rubia sikkimensis* [33], and* Rubia tetragona* [32]
30	Quinizarin (1,4-dihydroxy-AQ)	OH	H	H	OH	H	H	H	H	*Rubia cordifolia* [32] and* Rubia tinctorum* [32]
31	1,4-Dihydroxy-2-(hydroxymethyl)-AQ	OH	HOCH_2_	H	OH	H	H	H	H	*Rubia cordifolia* [32] and* Rubia yunnanensis* [32]
32	2-(Ethoxycarbonyl)-1,4-dihydroxy-AQ	OH	EtOOC	H	OH	H	H	H	H	*Rubia cordifolia* [32]
33	Christophine (2-(ethoxymethyl)- 1,4-dihydroxy-AQ)	OH	EtOCH_2_	H	OH	H	H	H	H	*Rubia tinctorum* [32]
34	1,4-Dihydroxy-2-methyl-AQ	OH	Me	H	OH	H	H	H	H	*Rubia cordifolia* [32] and *Rubia lanceolata* [32]
35	Xanthopurpurin (1,3-dihydroxy-AQ)	OH	H	OH	H	H	H	H	H	*Rubia tinctorum* [32]*, Rubia cordifolia* [32]*, Rubia oncotricha* [39]*, *and* Rubia yunnanensis* [43],
36	Xanthopurpurin 3-methyl ether (1-hydroxy-3-methoxy-AQ)	OH	H	MeO	H	H	H	H	H	*Rubia tinctorum* [32]
37	Xanthopurpurin dimethyl ether (1,3-dimethoxy-AQ)	MeO	H	MeO	H	H	H	H	H	*Rubia tinctorum* [32]
38	1-Hydroxy-3-(methoxycarbonyl)-AQ	OH	H	MeOOC	H	H	H	H	H	*Rubia tinctorum* [32], *Rubia lanceolata* [39], and *Rubia oncotricha* [39]
39	Pseudopurpurin (3-(carboxy)-1,2,4-trihydroxy-AQ)	OH	OH	HOOC	OH	H	H	H	H	*Rubia tinctorum* [32]*, Rubia cordifolia* [32]*, *and* Rubia peregrine* [32]
40	1,4-Dihydroxy-2-methyl-5-methoxy-AQ	OH	Me	H	OH	MeO	H	H	H	*Rubia cordifolia* [50]
41	1,4-Dihydroxy-2-methyl-8-methoxy-AQ	OH	Me	H	OH	H	H	H	MeO	*Rubia cordifolia* [50]
42	1,4-Dihydroxy-6-methyl-AQ	OH	H	H	OH	H	Me	H	H	*Rubia cordifolia* [51]
43	1,5-Dihydroxy-2-methyl-AQ	OH	Me	H	H	OH	H	H	H	*Rubia cordifolia* [51]
44	Physcion (1,8-dihydroxy-3-methoxy-6-methyl-AQ)	OH	H	Me	H	H	Me	H	OH	*Rubia cordifolia* [50] and* Fallopia japonica* [52]
45	2-Methyl-1,3,6-trihydroxy-AQ	OH	Me	OH	H	H	OH	H	H	*Rubia cordifolia* [32]*, Rubia sylvatica* [32]*, Rubia yunnanensis* [43], *Rubia lanceolata* [32], and* Rubia schumanniana* [32]
46	1,4-Dihydroxy-7-methyl-AQ	OH	H	H	OH	H	H	Me	H	*Rubia cordifolia* [50]
47	4,5-Dihydroxy-2-methoxy-7-methyl-AQ	H	Me	H	OH	OH	H	MeO	H	*Rubia cordifolia* [50]
48	2,7-Dihydroxy-4-methoxy-3-methyl-AQ	H	OH	Me	MeO	H	H	OH	H	*Rubia yunnanensis* [32]
49	2-Hydroxy-7-methyl-AQ	H	Me	H	H	H	H	OH	H	*Rubia tinctorum* [32]
50	2-Carboxy-4-hydroxy-AQ	H	HOOC	H	OH	H	H	H	H	*Rubia cordifolia* [32]
51	3-(*β*-D-Glucopyranosyloxy)-1,6-dihydroxy-2-methyl-AQ	OH	Me	GluO	H	H	OH	H	H	*Rubia cordifolia* [32]
52	3-(6-O-Acetyl-*β*-D-glucopyranosyloxy)-1,6-dihydroxy-2-methyl-AQ	OH	Me	6-OAc- GluO	H	H	OH	H	H	*Rubia cordifolia* [32]
53	3-[(2-O-6-Deoxy-*α*-L-mannopyranosyl-*β*-D-glucopyranosyl)oxy]-1,6-dihydroxy-2-methyl-AQ	OH	Me	6-dManO-GluO	H	H	OH	H	H	*Rubia cordifolia* [32], *Rubia schumanniana* [32]*, Rubia akane* [39],* *and* Rubia yunnanensis* [43]
54	3-[(3-O-Acetyl-2-O-6-deoxy-*β*-D-mannopyranosyl-*β*-D-glucopyranosyl)oxy]-1,6-dihydroxy-2-methyl-AQ	OH	Me	3-OAc- 6-dManO-GluO	H	H	OH	H	H	*Rubia cordifolia* [32]
55	3-[(6-O-Acetyl-2-O-6-deoxy-*β*-D-mannopyranosyl-*β*-D-glucopyranosyl)oxy]-1,6-dihydroxy-2-methyl-AQ	OH	Me	6-OAc- 6-dManO-GluO	H	H	OH	H	H	*Rubia cordifolia* [32]*, Rubia akane* [39],* Rubia yunnanensis* [43]*, *and* Rubia schumanniana* [32]
56	3-[(3,6-O-Diacetyl-2-O-6-deoxy-*β*-D-mannopyranosyl-*β*-D-glucopyranosyl)oxy]-1,6-dihydroxy-2-methyl-AQ	OH	Me	3,6-[OAc]_2_-6-dManO- GluO	H	H	OH	H	H	*Rubia cordifolia* [32]
57	3-[(4,6-O-Diacetyl-2-O-6-deoxy-*β*-D-mannopyranosyl-*β*-D-glucopyranosyl)oxy]-1,6-dihydroxy-2-methyl-AQ	OH	Me	4,6-[OAc]_2_-6-dManO- GluO	H	H	OH	H	H	*Rubia cordifolia* [32]
58	3-[(4-O-Acetyl-2-O-6-deoxy-*β*-D-mannopyranosyl-*β*-D-glucopyranosyl)oxy]-1,6-dihydroxy-2-methyl-AQ	OH	Me	4-OAc-6- dManO- GluO	H	H	OH	H	H	*Rubia cordifolia* [32]
59	3-[(6-O-Acetyl-2-O-*β*-D-xylopyranosyl-*β*-D-glucopyranosyl)oxy]-1,6-dihydroxy-2-methyl-AQ	OH	Me	6-OAc- XylO- GluO	H	H	OH	H	H	*Rubia cordifolia* [32]
60	Ruberythric acid (1-hydroxy-2-[(6-O-*β*-D-xylopyranosyl-*β*-D-glucopyranosyl)oxy]-AQ)	OH	XylO- GluO	H	H	H	OH	H	H	*Rubia cordifolia* [32]*, Rubia tinctorum* [32], and * Rubia iberica* [45]
61	Lucidin primeveroside (1-hydroxy-2-(hydroxymethyl)-3-[(6-O-*β*-D-xylopyranosyl-*β*-D-glucopyranosyl)oxy]-AQ)	OH	HOCH_2_	XylO- GluO	H	H	OH	H	H	*Rubia cordifolia* [32]*, Rubia tinctorum* [32]*, Rubia iberica* [45], and *Rubia yunnanensis *[43]
62	1-Acetyl-3-[(4-O-6-deoxy-*β*-D-mannopyranosyl-*β*-D-glucopyranosyl)oxy]-6-hydroxy-2-methyl-AQ	MeCO	Me	6-dManO-GluO	H	H	OH	H	H	*Rubia cordifolia* [32]
63	2-[(6-O-*β*-D-Glucopyranosyl-*β*-D-glucopyranosyl)oxy]methyl-11-hydroxy-AQ	H	H	H	H	H	H	H	GluO-GluO	*Rubia cordifolia* [32] and* Rubia schumanniana* [32]
64	3-[(2-O-6-Deoxy-*β*-D-mannopyranosyl-*β*-D-glucopyranosyl)oxy]-1-hydroxy-2-(methoxycarbonyl)-AQ	OH	MeOOC	6-dManO-GluO	H	H	H	H	H	*Rubia cordifolia* [32]
65	3-(*β*-D-Glucopyranosyloxy)-2-(hydroxymethyl)-AQ	H	HOCH_2_	GluO	H	H	H	H	H	*Rubia tinctorum* [32]
66	3-(*β*-D-Glucopyranosyloxy)-8-hydroxy-2-(hydroxymethyl)-AQ	H	HOCH_2_	GluO	H	H	H	H	OH	*Rubia tinctorum* [32]
67	2-(*β*-D-Glucopyranosyloxy)-1,3-dihydroxy-AQ	OH	GluO	OH	H	H	H	H	H	*Rubia tinctorum* [32]
68	3-(*β*-D-Glucopyranosyloxy)-1-hydroxy-2-(hydroxymethyl)-AQ	OH	HOCH_2_	GluO	H	H	H	H	H	*Rubia cordifolia* [32]
69	Emodin (1,3,8-trihydroxy-6-methyl-AQ)	OH	H	Me	H	H	OH	H	OH	*F. japonica* [28, 52]
70	Cascarin (emodin 6-O-rhamnoside)	OH	H	Me	H	H	RhaO	H	OH	*Rhamnus *sp. [28]
71	Rhein (1,8-dihydroxy-3-carboxyl-AQ)	OH	H	HOOC	H	H	H	H	OH	*Cassia*sp. [28]
72	Catenarin (1,4,6,8-tetrahydroxy-3-methyl-AQ)	OH	H	Me	OH	H	OH	H	OH	*Helminthosporium catenarium* [28]
73	Aloe-emodin (1,8-dihydroxy 3-hydroxy methyl anthraquinone)	OH	H	CH_2_OH	H	H	H	H	OH	*Aloe vera* [53], *Cassia*sp. [53], *Rhamnus frangula,*Cascara Sagrada* *[53]*, Rhamnus purshiana* [53], and* Rheum rhaponticum* [53]
74	Chrysophanol (1,8-dihydroxy-3-methyl-AQ)	OH	H	Me	H	H	MeO	H	OH	*Cassia *sp. [54]
75	Rhein-8-glucoside	OH	H	HOOC	H	H	H	H	GluO	*Cassia *sp. [54]
76	Alatinone (1,5,7-trihydroxy-3-methyl-AQ)	OH	H	Me	H	OH	H	OH	H	*Cassia *sp. [54]
77	Diacerein (diacerhein)	OAc	H	HOOC	H	H	H	H	OAc	*Cassia *sp. [54]
78	Fistulic acid	OH	Me	HOOC	OH	H	MeO	MeO	OH	*Cassia *sp. [54]
79	5-Hydroxy emodin	OH	H	Me	H	OH	OH	H	OH	*Cassia *sp. [54]
80	1,3-hihydroxy-6,8-dimethoxy-AQ	OH	H	OH	H	H	MeO	H	MeO	*Cassia *sp. [54]
81	1,3,5,8-Tetrahydroxy-2-methyl-AQ	OH	Me	OH	H	OH	MeO	H	OH	*Cassia *sp. [54]
82	1,2-Dihydro-1,3,8-trihydroxy-2-methyl-AQ	OH	Me	OH	H	H	H	H	OH	*Cassia *sp. [54]
83	1,8-Dihydroxy-6-methoxy-2-methyl-AQ	OH	Me	H	H	H	MeO	H	OH	*Cassia *sp. [54]
84	1,8-Dihydroxy-6-methoxy-3-methyl-AQ	OH	H	Me	H	H	MeO	H	OH	*Cassia *sp. [54]
85	Citreorosein (1,3,8-trihydroxy-6-hydroxymethyl-AQ)	OH	H	CH_2_OH	H	H	OH	H	OH	*Cassia *sp. [54]
86	Emodic acid (1,6,8-trihydroxy-AQ-3-carboxylic acid)	OH	H	HOOC	H	H	OH	H	OH	*Cassia *sp. [54]
87	Obtusifolin (2,8-dihydroxy-1-methoxy-3-methyl-AQ)	MeO	OH	Me	H	H	H	H	OH	*Cassia *sp. [54]
88	2-Formyl-1,3,8-trihydroxy-AQ	OH	CHO	OH	H	H	H	H	OH	*Cassia *sp. [54]
89	3-Formyl-1-hydroxy-8-methoxy-AQ	OH	H	CHO	H	H	H	H	MeO	*Cassia *sp. [54]

Glu: glucosyl; dMan: deoxymannosyl; Rha: rhamnosyl; Xyl: xylosyl; Me: methyl; Et: ethyl; Ph: phenyl; Ac: acetyl.

**Table 2:** NOAQs with antidiabetic activities.

S. number	Name	Classification	Molecular formula	Biological activities
72	Catenarin	Anthraquinone	C_15_H_10_O_6_	Antichemotactic [28] and antidiabetic [28]
69	Emodin	Anthraquinone	C_15_H_10_O_5_	Antichemotactic [67] and antidiabetic [67]
44	Physcion	Anthraquinone	C_16_H_12_O_5_	Antichemotactic [28]
70	Cascarin	Anthraquinone	C_21_H_20_O_9_	Antichemotactic [28]
71	Rhein	Anthraquinone	C_15_H_8_O_6_	Antichemotactic [28]
77	Diacerein	Anthraquinone	C_19_H_12_O_8_	Antiosteoarthritic [68] and antidiabetic [28]

## Abbreviations

AID: Autoimmune diabetes
NOAQ: Naturally occurring anthraquinone
NOD mice: Nonobese diabetic mice
GPCR: G protein-coupled receptor
MAPK: Mitogen-activated protein kinase
MAPKK: Mitogen-activated protein kinase
IC_50_: Half maximal inhibitory concentration
STZ: Streptozotocin
BB rats: Biobreeding rats
LETL rats: Long Evans Tokushima Lean rats.
